# AFM Measurements and Testing Properties of HDPE and PBT Composites with Fillers in the Form of Montmorillonite and Aluminum Hydroxide

**DOI:** 10.3390/ma15248738

**Published:** 2022-12-07

**Authors:** Zbigniew Bałaga, Adam Gnatowski, Sławomir Kulesza, Mirosław Bramowicz, Monika Gwoździk

**Affiliations:** 1 Faculty of Production Engineering and Materials Technology, Czestochowa University of Technology, Armii Krajowej Street 19, 42-201 Czestochowa, Poland; 2 Faculty of Mechanical Engineering and Computer Science, Czestochowa University of Technology, Armii Krajowej Street 21, 42-201 Czestochowa, Poland; 3 Faculty of Technical Sciences, University of Warmia and Mazury in Olsztyn, Oczapowskiego 11, 10-719 Olsztyn, Poland

**Keywords:** high-density polyethylene (HDPE), polybutylene terephthalate (PBT), montmorillonite, aluminum hydroxide, polymer–matrix composites

## Abstract

This paper presents the effect of the addition of fillers such as aluminum hydroxide or montmorillonite on the structure and properties of polymers such as high-density polyethylene (HDPE) and polybutylene terephthalate (PBT). Both types of specimens were obtained by injection molding. X-ray diffraction examinations were performed on the materials obtained to determine the effect of the addition of the fillers used on the degree of crystallinity of the composites. The density and hardness of the composites were evaluated, and the static tensile test and the analysis of the structure parameters using atomic force microscopy (AFM) were also carried out. It was shown that the addition of powder fillers to polymers such as high-density polyethylene and polybutylene terephthalate affects the structure parameters such as surface roughness, mean grain size, anisotropy ratio, fractal dimension, the corner frequency of the composites, and mechanical properties such as Young’s pseudo-modulus, average adhesion force, hardness, and tensile strength.

## 1. Introduction

Processes of mixing polymers with fillers are used to obtain composite materials with specific mechanical, thermal, and structural properties. The volume of HDPE and PBT composites is on the rise among the polymer materials being produced today. This is justified by the numerous advantages of their manufacturing methods, the relative ease of mixing during processing, the extended possibilities of their use, and their operating conditions. The properties of the polymeric material products depend on both the type of filler used and the structural factors of the polymer. The structural factors include the molecular weight, the chemical structure of the macromolecules, the physical structure of the chain, crystallinity, and molecular orientation. Functional conditions are the temperature, load time, pressure, type of deformations, etc. [1,2,3,4].

Predicting properties and structure plays an important role in planning the composition and manufacturing of polymer products. The structure is very important as it determines the use of composites for manufacturing parts of machinery and equipment. HDPE is a material from the polyolefin group of hydrocarbon materials. It has good tribological properties with low specific mass. It is a very popular material used in many industries. Its applications are very wide including packaging in the food or cosmetics industries or the automotive sector [1,2,3]. Alan et al. [5] proposed the use of polymeric waste to make new building materials using fly ash.

The examinations were conducted using two kinds of typical fly ash, from coal to polyethylene bags. The tensile strength and chemical resistance of the materials were investigated. The characteristics of these materials were also studied by [5,6] using XRD, IR, and SEM techniques. The tensile strength of the materials ranged from 0.92 to 2.62 kp/mm^2^. Better results were reported when the proportion of fly ash was low. The composites obtained also displayed high chemical resistance, as reported by Shut in [6], who described the industrial advantages of using a filler composite. The advantages of using these and similar fillers have also been described in other publications. For example, compounds containing up to 50% fly ash were extruded and pressure molded into construction materials. Fillers of this type add rigidity and reduce costs. Compounds with a fly ash content of 15÷30% are under prototype testing for injection molded automotive trims. In addition to adding rigidity, fly ash increases the recycling fraction of automotive parts. In order to improve the interaction between the microsphere filler and the matrix, the authors of the study in [7] treated the surface with a silane coupling agent and HDPE-g-dibutyl maleate as a compatibilizer. The tensile strengths and thermal properties of the composites were evaluated. The results showed that both surface modification and compatibilization led to significant improvements in the mechanical properties and thermal stability of the composites. Similar examinations [8,9,10,11] have been presented by authors investigating the effect of filler on changes in the mechanical properties of polyethylene. These authors used a worm extruder to obtain composites whereas injection molding was employed to obtain the specimens. The obtained specimens were examined for their microstructure using scanning electron microscopy and changes in the mechanical properties were evaluated. The present study examined HDPE composites with montmorillonite, which is present in clay rocks (mainly bentonite). However, it is essential that fossil montmorillonite has to be modified. The nanocomposite undergoes a purification treatment until it reaches the content of components with a nonlamellar structure below 5% by volume. With the high ion-exchange index and high specific surface area of the nanoparticles, researchers have been increasingly interested in this material as a filler for polymer nanocomposites [12,13]. This material contains three-layer packages. These packages are composed of a single octahedral and two tetrahedral layers. Between the packages are metal cations (mainly sodium and calcium) and water molecules. The resulting montmorillonite nanoparticles are subjected to hydrophobization to increase its compatibility with polymers. This phenomenon primarily involves the exchange of metal cations for organic cations. These processes lead to obtaining modified montmorillonite. With the increased distance between packages of montmorillonite obtained through modification, it is possible to ensure easier penetration of the monomer and polymer particles into the spaces between the layers. The montmorillonite modification processes improve its hydrophobicity and increase its versatility as a filler for use with various polymers [12,13,14]. Another polymer used in the study is polybutylene terephthalate (PBT), which belongs to the group of partially crystalline polymers and is characterized by high molecular weight and good processing properties.

PBT is a material with high chemical resistance at room temperature. It is resistant to exposure to a wide range of chemical compounds such as fats, gasoline, aliphatic hydrocarbons, oils, glycol, alcohol, and low-concentration bases and acids [15,16,17]. The aluminum hydroxide employed in the study was an inorganic agent used as a filler in composites designed to reduce flammability. An aluminum hydroxide addition of 50 to 65% by weight is usually used to reduce the polymers’ propensity to ignite [18]. However, the addition of a flame retardant with such concentrations was found to adversely affect the mechanical properties of the composite compared to the pure polymer. Due to aluminum hydroxide’s decomposition temperature of more than 190 °C, it has been used to modify polycarbonates, polyesters, polyethylene terephthalate, polybutylene terephthalate, and polyamides [18,19,20,21,22]. The aim of the present study was to evaluate the effect of fillers on the selected properties and morphology of the HDPE and PBT structures. The paper presents the results of the evaluation of the mechanical properties and structural parameters determined by atomic force microscopy. In the current literature, it is difficult to find similar studies of polymeric materials or composites based on them, which have attempted to describe the structure with fractal parameters or determine nanomechanical properties such as Young’s pseudo-modulus or adhesion forces.

## 2. Materials and Methods

The study examined polymer composites with HDPE (high-density polyethylene) and PBT (polybutylene terephthalate) used as a matrix. Specimens were obtained by injection molding, with montmorillonite with a content of 3 and 7 wt% used as a filler for HDPE, while in the case of PBT, the filler was aluminum hydroxide with a content of 3 and 7 wt%.

The specimens were subjected to X-ray diffraction using the radiation of a cobalt anode lamp, then the density was determined by the Archimedes method using a hydrostatic balance, and the hardness was measured using two methods, the Shore method and the ball method, and the static tensile test was performed. The final stage was atomic force microscopy (AFM) examinations, aimed to evaluate the surface parameters in the specimens studied. Table 1 shows the chemical composition of the specimens used in the paper.

The examinations were conducted with high-density polyethylene (HDPE) (Hostalen GC 7260, manufactured by LyondellBasell Industries Holdings, Rotterdam, The Netherlands) used for injection molding. The filler was Closite SE 3010 montmorillonite (BYK Additives Inc., Wesel, Germany).Before processing, the polyethylene was prepared by drying it using a Zelmet device with a KC-100/200 heating chamber. Drying was carried out at 70 °C, and the drying time did not exceed 4 h. Preparation with the silane compound montmorillonite was used. The components were mixed in a plasticized state and extruded. The test specimens (in the form of standardized tensile test specimens) were injected using a Krauss Maffei injection molding machine (KM65-160 C4) with a mold closing force of 650 kN, equipped with a feed screw with a diameter of 30 mm and a ratio of L/D = 23. The following injection molding process parameters were used to achieve the optimal properties of the specimens:

- Injection nozzle temperature: 205 °C;
- Mold cavity temperature: 40 °C;
- Injection rate: 45 cm^3^/s;
- Injection time: 0.45 s;
- Injection pressure: 60 MPa;
- Clamping pressure: 30 MPa;
- Holding time: 28 s;
- Cooling time: 15 s.

Another test material was polybutylene terephthalate (PBT, Crastin 6130 NC010; manufactured by DuPont, Wilmington, DE, USA). Tests were conducted for specimens made of aluminum hydroxide (ATH) with polybutylene terephthalate (Apyral 40CD; manufacturer Nabaltec, Schwandorf, Germany). The composite was prepared for processing by drying in a Shini CD Cabinet dryer at 80 °C for 12 h. The components were mixed in a viscoplastic state using a screw extruder with a screw diameter of 30 mm and a length-to-diameter ratio of 27. A rotor mill was used to granulate the extruded product. A Krauss Maffei KM65-160C1 injection molding machine was employed to obtain molded pieces, with a clamping force of 650 kN and a 30 mm diameter screw, the length-to-diameter ratio of 23, three zones, and a constant pitch along its length. The injection molding parameters used to ensure the optimal properties of the specimens were as follows:

- Nozzle temperature: 260 °C;
- Mold temperature: 80 °C;
- Injection rate: 45 cm^3^/s;
- Injection time: 0.45 s;
- Injection pressure: 130 MPa;
- Clamping pressure: 80 MPa;
- Holding time: 20 s;
- Cooling time: 25 s.

**Figure 1:**
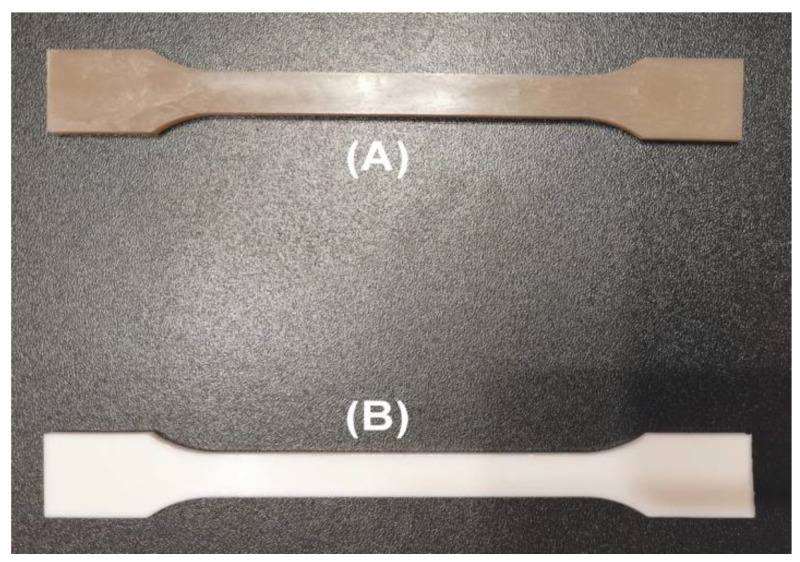
Examples of the composite samples produced in the injection process: (**A**) composite with HDPE matrix, (**B**) composite with PBT matrix.

The shape of the samples was in accordance with the PN-EN ISO 527-1:2020-01 standard for the polymer samples used for static tensile testing. Examples of the composite samples produced are shown in Figure 1.

## 3. Results and Discussion

**Figure 2:**
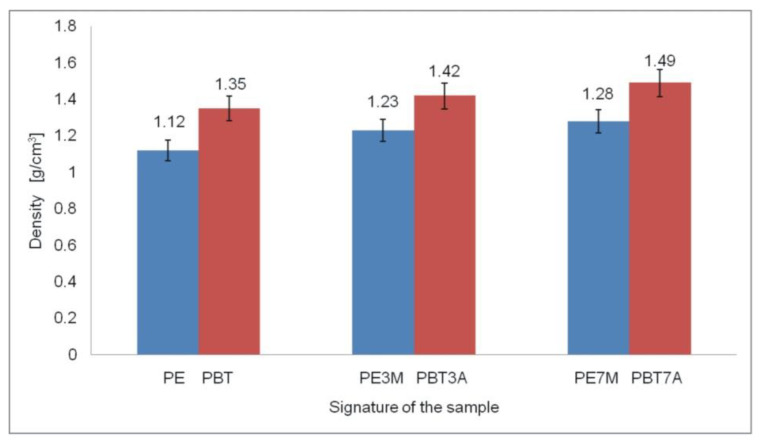
Density of the materials obtained.

Density was tested using the hydrostatic method in accordance with PN-EN ISO 1183-1:2019-05. The results showed that the addition of fillers such as montmorillonite to HDPE and aluminum hydroxide to PBT caused a slight increase in the density of the materials obtained. An increase in the density of both composites was observed with an increase in the amount of fillers in the polymer matrix, which resulted from the fact that the added montmorillonite and aluminum hydroxide had a higher density than the HDPE and PBT polymers. The results obtained are presented in Figure 2.

X-ray phase analysis showed that the materials obtained were characterized by an amorphous-crystalline structure. Such a nature is determined by the very structure of the polymers used as a matrix in the materials tested. According to the formula proposed by Wunderlich [22], the mass fraction of crystalline phases is directly proportional to the X-ray intensity in crystalline regions whereas the diffusely scattered X-ray intensity is proportional to the mass fraction of the amorphous phase. The degree of crystallinity of the specimens was determined based on the diffraction data. Therefore, the reflections originating from the crystalline phases and the wide-angle reflection originating from the amorphous phase were separated on the obtained diffraction images using the pseudo-Voigt function.

(1) $$Sk=\frac{\sum I_{kryst}}{K\cdot I_{amorf}+\sum I_{kryst}}$$

where

$\sum I_{kryst}$—the sum of the integral intensities of radiation in the crystalline regions;
$I_{amorf}$—integral intensity of diffusely-scattered radiation in amorphous regions;
K—constant.

**Figure 3:**
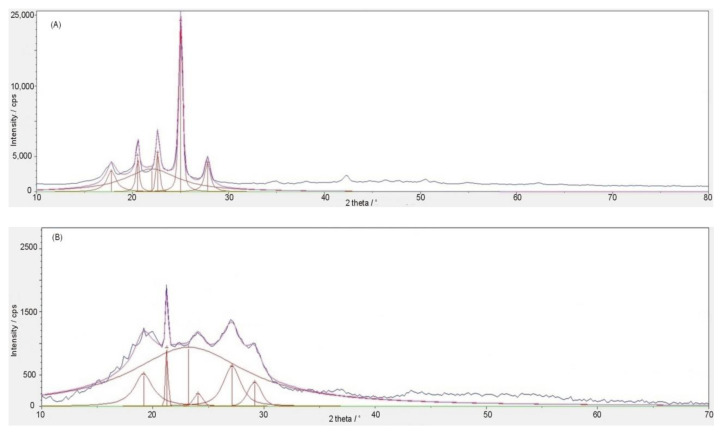
Examples of the X-ray diffractions with resolved diffraction reflections originating from the crystalline and amorphous phases: (**A**) PE7M; (**B**) PBT7A.

Examples of the effects of using the pseudo-Voigt function to resolve the individual diffraction reflections for the PBT- and HDPE-based specimens are shown in Figure 3.

It is noteworthy that all of the diffractions obtained for both PBT-based specimens regardless of the addition of aluminum hydroxide Al(OH)_3_ and HDPE-based specimens regardless of the addition of montmorillonite Al_2_O_3_·SiO_2_·nH_2_O were similar in nature in their groups. The presence of diffraction reflections originating from the polymers constituting the matrix was found, whereas reflections originating from the fillers used were not observed. The determined degrees of crystallinity in individual groups of specimens varied slightly. These were ca. 90% for the polyethylene-based specimens and 70% for the polybutylene terephthalate-based specimens. These values were similar to those obtained for the pure polymers.

**Figure 4:**
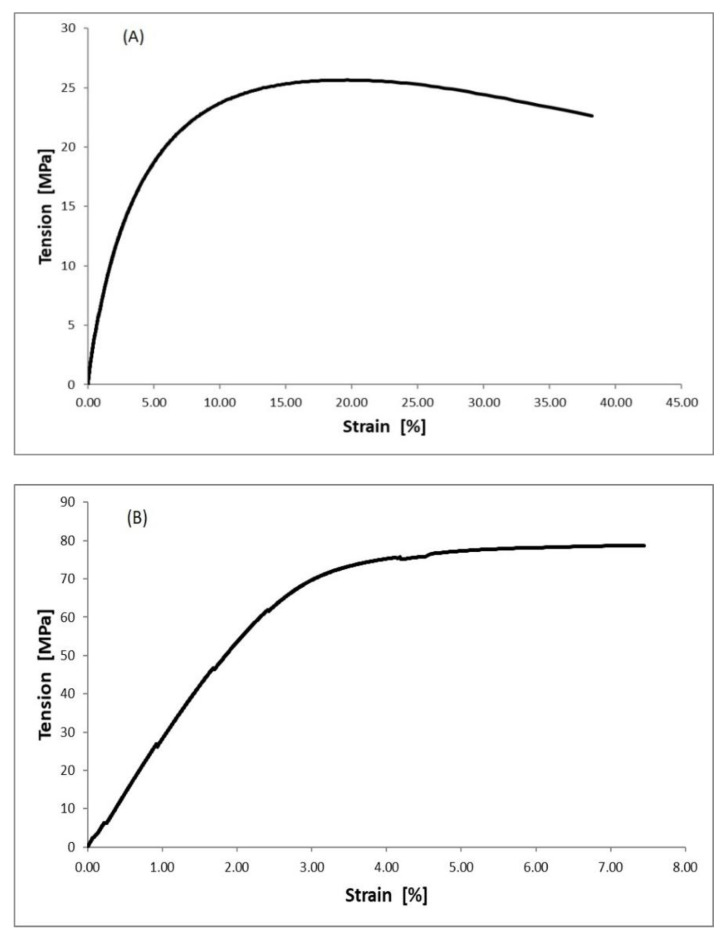
Examples of tensile strength curves for: (**A**) PE7M; (**B**) PBT7A.

A static tensile test was carried out in accordance with PN-EN ISO 527-1:2020-01 to determine the mechanical properties of the specimens. An example of the tensile strength curves for the selected PBT and HDPE matrix specimens is shown in Figure 4.

**Figure 5:**
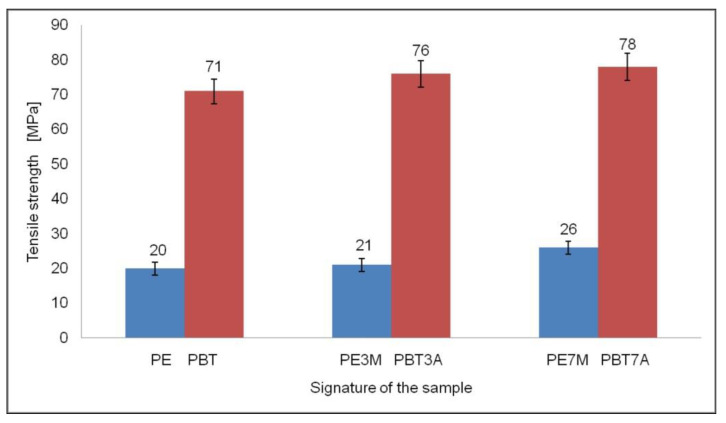
Tensile strength results.

As can be seen from Figure 5, the addition of montmorillonite fillers to high-density polyethylene (HDPE) in amounts of 3 and 7% and aluminum hydroxide to polybutylene terephthalate in the same amounts resulted in both cases showing a very slight increase in tensile strength. For the HDPE-matrix specimens, these values ranged from 20 to 26 MPa, while for the PBT-matrix specimens, these were from 71 to 78 MPa.

**Figure 6:**
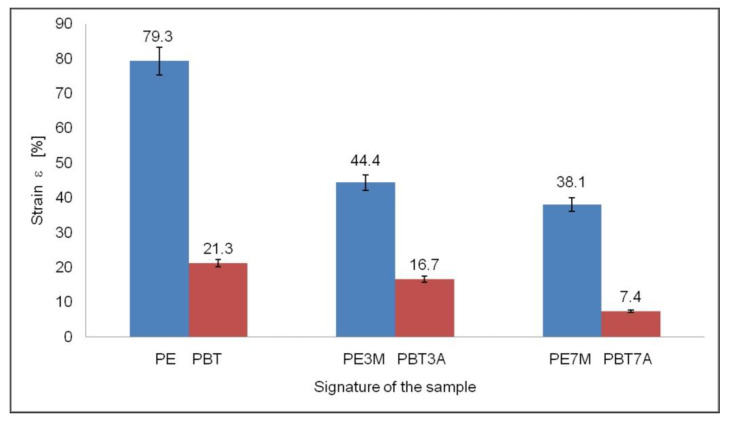
Specimen strain.

A much greater effect of filler addition was observed for the strain (Figure 6). For the HDPE-matrix specimen, it was found that the strain decreased from ca. 79% for pure HDPE to ca. 44% for the specimen with the addition of 3% montmorillonite and ca. 38% for the specimen where the montmorillonite content was 7 wt%. Similar dependencies were found for the effect of the addition of aluminum hydroxide on the strain of PBT. This decreased from ca. 21% for pure PBT to about 16% for the specimen with 3 wt% of filler and 7% for the specimen with 7 wt% of filler phase.

The tensile strength and strain values for each specimen are shown in Figure 5 and Figure 6.

In order to estimate the effect of the filler additives used on the hardness of the specimens, measurements were carried out using the Shore method in accordance with PN-EN ISO 868:2005 and the ball pressing method in accordance with PN-EN ISO 2039-1:2004. Ten measurements were made on each sample, with the mean values of the hardness shown in Table 2.

Both in the case of the tensile strength and hardness measurements, it was difficult to determine the mechanisms leading to a slight increase in these properties in the composites. This effect was probably due to the good dispersion of the fillers particles used in the composite matrix and good adhesion at the particle–matrix interface. In the case of hardness, the impact of the particle surface on the matrix can lead to its strengthening, while in the case of the static tensile test, these particles, up to a certain level of stresses, can be an obstacle to the progression of micro-cracks in the matrix formed during the stretching process.

Atomic force microscopy (AFM) and scanning electron microscopy are used to evaluate surface topography and fractal analysis [23,24,25,26,27,28,29,30,31,32]. Atomic force microscopy examinations were performed to describe the surface topography of the specimens.

Specific surface morphology of the composite films in this study was examined using the atomic force microscopy (AFM) method in the peak force tapping mode (Bruker). When scanning, the vibrating cantilever repeatedly follows a sinusoidal approach-withdraw path in order to grab a series of force-distance curves. Assuming that the peak force(i.e., the maximum temporary repulsion force) remains constant, not only the surface topography but also several material properties can be extracted including Young’s pseudo-modulus, adhesion forces, etc. The images were recorded using a HQ:NSC14/Al BS (MikroMasch) silicon probe with a nominal tip radius of 8 nm attached to a rectangular cantilever with a force constant of 5 N/m and resonance frequency of 160 kHz. The peak force was 500 pN throughout the measurements.

**Figure 7:**
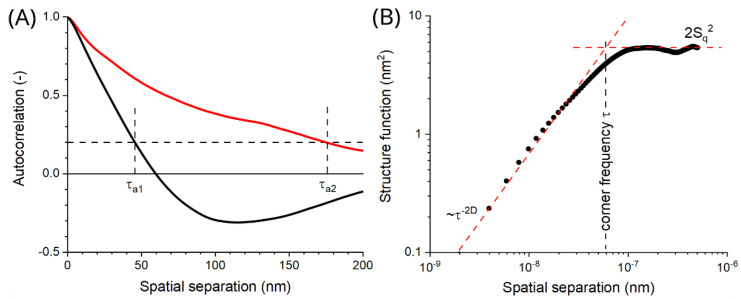
Example curves derived from the AFM images of the composite films that are helpful in obtaining spatial characteristics of their complex surface geometry: (**A**) Profile plots of the autocorrelation function along with directions of its extreme decay lengths: *τ_a_*_1_ and *τ_a_*_2_, respectively; (**B**) double-log structure function with the threshold of the allometric scaling behavior–corner frequency *τ*—marked with the dashed line.

In order to derive the specific spatial characteristics of the surface texture, several methods were joined together. In the first step, the 2-dimensional autocorrelation function was calculated [33]:

(2) $$R_{mn}=\frac{1}{\left(N-n)(N-m\right)}\sum_{P=1}^{N-n}\sum_{q=1}^{N-m}(z_{p+m,q+n}*z_{p,q})$$

where (*m*, *n*) integers are coordinates of samples in AFM images. With increasing lateral separation, autocorrelation gradually decays to zero, which serves as a measure of orientation-dependent changes in the surface texture characteristics referred to as the texture anisotropy ratio *S_tr_* [33]:

(3) $$S_{tr}=\frac{\tau_{a1}}{\tau_{a2}}$$

where *a*_1_, and *a*_2_ are the directions of the fastest and the slowest autocorrelation decay down to a value of 0.2, respectively, as shown in Figure 7A.

In order to step into multi-scale characterization that stems from the scale-invariance of the spatial features, the structure function needs to be computed according to:

(4) $$S_{mn}=2S_{q}^{2}(1-R_{mn})$$

where *S_q_* is the surface roughness (RMS). Figure 7B shows that the profile structure function averaged around its origin exhibits a specific allometric dependence upon the wavelength [34,35]:

(5) $$S(\tau )=K\tau^{2(2-D)}$$

where *K* is the pseudo-topothesy, and *D* is the fractal dimension. In general, *D* and *K* correspond to the way how the amplitudes of surface height variations (relative and absolute, respectively) scale with the wavelengths.

Allometricity stays true until the threshold (referred to as the corner frequency τ) is approached. Beyond that point, the plot asymptotically goes flat at the level of 2*S_q_*^2^. The presence of several thresholds in the structure function is known as multifractality, which is associated with possible higher-order alignments among geometric sub-units on the surface studied.

**Figure 8:**
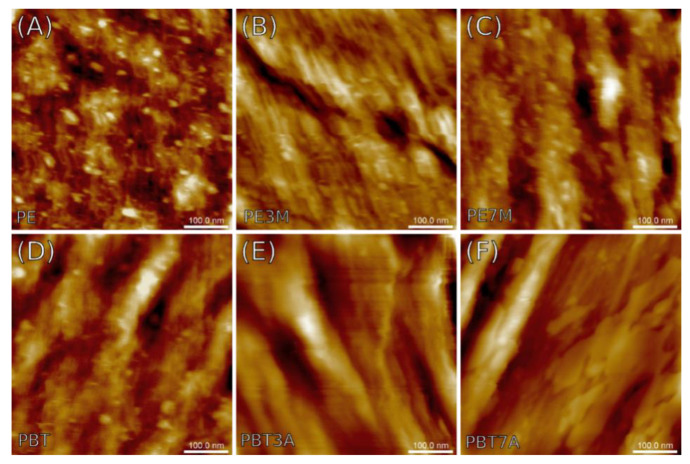
The AFM topographical images of the composites taken from metallographic microsections. Samples: (**A**) PE; (**B**) PE3M; (**C**) PE7M; (**D**) PBT; (**E**) PBT3A; (**F**) PBT7A.

**Figure 9:**
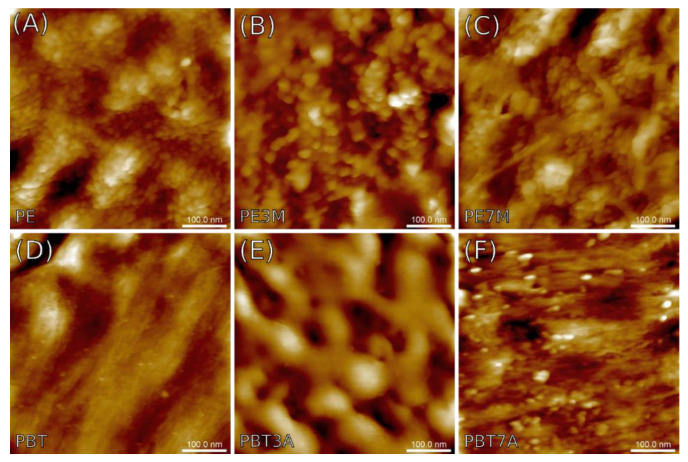
The AFM topographical images of the composites under study taken from the fracture cross-sections. Samples: (**A**) PE; (**B**) PE3M; (**C**) PE7M; (**D**) PBT; (**E**) PBT3A; (**F**) PBT7A.

Figure 8 and Figure 9 show the AFM topographical images of the surfaces of the specimens studied recorded on the metallographic and breakage sections, respectively. In addition to surface topography, the AFM measurements also yielded maps of the nanomechanical properties of the composite materials concerning the Young’s pseudo-modulus and adhesion forces. The results are summarized in Table 3 and Table 4 respectively.

Figure 8A exhibits quite a flat surface of the polyethylene reference sample, covered with small, spherical precipitates around 20 nm in diameter and areal concentration approaching 10^11^ cm^−2^. Similar features can be seen on polyethylene surfaces with certain amounts of montmorillonite added to the structure, which are shown in Figure 8B,C. Unlike the reference, however, the two remaining specimens, that is, PE3M and PE7M, revealed the presence of long-wavelength components in the form of parallel longitudinal grooves separated around 150 nm from each other, contributing to the surface waviness. Figure 8D,E demonstrates a series of similar grooves that appeared in the AFM images of specimens made of polybutylene terephthalate (PBT). Compared to previous images, however, additional spherical precipitates occurred only in the reference sample (pure PBT), although more diversified in size and with lesser areal concentration (ca. 10^10^ cm^−2^).

Various characteristics of the surface variability seen in metallographic microsections are generally consistent with the conclusions drawn from the visual examination of the AFM images presented in Figure 8. First of all, the RMS value of the surface roughness *S_q_* was the lowest in the pure PE sample, but the largest in both PBT samples enriched with aluminum hydroxide. Diameters of the precipitates previously estimated from Figure 8 agreed well with the average grain diameters *d_mean_* shown in Table 3. The mean values varied between 13 and 20 nm, whereas the areal concentrations remained somewhere in the range between 10^10^ and 10^11^ cm^−2^. The surface anisotropy ratio *S_tr_* was less than 0.51 (maximum found for PE3M sample), a value specific of surfaces with a substantial dependence of the surface texture characteristics on the observation axis.

Scale-invariant spatial characteristics expressed in terms of fractal parameters such as fractal dimension *D* and corner frequency *τ* give insights into the similarities between the surface components of various sizes. That said, all surfaces exposed using metallographic microsections can be described using single parameters referred to as monofractality, which provide the clue for short-range order patterns without any secondary spatial correlation between topographical entities (aggregation). Fractal dimensions varied between 2.33 and 2.47 as in less-developed surfaces, while the corner frequency varying from 50 to 80 nm established the upper limit for the scaling behavior. Note that the limits were larger by a factor of 3–4 than the mean diameters of the precipitates, which might be at least partly related to their spatial alignment.

The parameters evaluated using nanomechanical mapping reflect the elasticity of the subsurface material and surface retention properties of a capillary layer on the surface. The mean Young’s pseudo-modulus obtained from four samples ranged from ca. 440 to 580 MPa, but for the PBT and PBT3A samples, it significantly rose to 780 or even 1110 MPa, respectively. For comparison, Amjadi and Fatemi [36] found that the elastic modulus of the pure HDPE samples made by injection molding varied between 600 and 800 MPa, depending on the profile thickness. A recent study by Saleh et al. [37] reported a modulus of ca. 950 MPa. In the case of PBT, the range of the results reported for the elastic modulus was even greater: from 2.3 GPa obtained by Hamlaoui et al. [38] up to 8 GPa reported by Borges et al. [39]. It should be noted, however, that published data (averaged at the macroscopic level) cannot be directly related to those obtained using nanomechanical mapping due to the non-homogeneity of the samples observed at the nanoscale. For surface adhesion, most samples yielded a tip-surface force between 1.6 and 2.2 nN, except for increased adhesion in PBT3A at ca. 3.4 nN.

Figure 9 presents the AFM topographical images of the surfaces of the cross-sectional views of the composite materials exposed using the breakage method. As a rule, the surfaces were covered with small precipitates except for the PBT3A specimen, showing a lack of small-scale objects. The mean size of these particles varied from several up to fifty nanometers and they appeared to form closed films on the surface of PE-based specimens, but on the other hand, they are loosely packed on the surface of the PBT-based composites. Apart from that, the image of the PE7M sample also exhibited the appearance of oblong objects around 200–300 nm long and a high aspect ratio, while that of PBT3A revealed a medium-range-order alignment of spherical structures 100 nm in their diameter, separated ca. 100 nm from each other in the nodes of a 2-dimensional array of 5- or 6-fold symmetry.

Table 4 presents the parameters describing specific aspects of height variations of the surfaces shown in Figure 9 (AFM images of cross-sectional views of the composite samples). Despite the same chemical composition as those in the microsections shown in Figure 7, the nanostructural properties of cross-sections make them different in several aspects compared to the data in Table 3. Some of these differences, for example, surface roughness, are likely to be caused by the preparation treatment for AFM imaging (room-temperature polishing vs. breakage), but others such as adhesion force might be associated with structural modifications of the material that occurred during the production process (heat transfer gradients, etc.).

The surface roughness *S_q_* of the cross-sections was found to vary between ca. 2.6 and 8.2 nm, which is generally larger compared to microsections. A similar observation was also made for the anisotropy ratio *S_tr_* (found to range from 0.37 to 0.73), which means a smaller sensitivity of surface texture characteristics upon the observation axis. As a rule, fractured cross-sections turn out to be much more isotropic than the metallographic microsections, which can lead to the conclusion that linear alignment or tubular structurization of the subunits observed on the surface was more prominent in the microsections than in the cross-sections.

Fractal parameters exhibited surfaces without any indications of agglomeration processes except for the PBT7A specimen, in which bifractal characteristics were observed. The fractal dimension in the monofractal samples fluctuated within the narrow range from 2.39 to 2.44 (2.33–2.47 in microsections), which shows specific homogeneity of the scaling behavior, and might be a result of the preparatory treatment (breaking). This agrees well with similar results obtained for the corner frequency, which was found to range from 52 to 69 nm. However, even though the corner frequency range appeared to be narrower than that for the microsections, both sets of samples yielded the same relative length of the corner frequency, being 3–4 times larger than the diameter of the precipitates seen on the surfaces.

**Figure 10:**
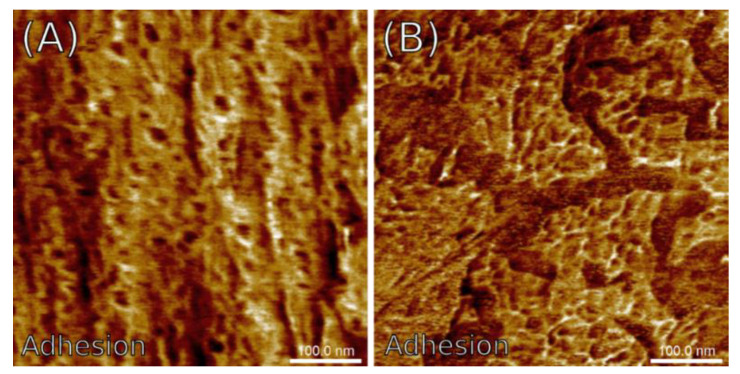
Exemplary maps of the adhesion force recorded in the PE7M sample: (**A**) microsection; (**B**) cross-section.

The average values of nanomechanical properties taken from cross-sectional AFM images were generally smaller compared to the microsections. The Young’s modulus *Y_mean_* varied between 170 and 620 MPa, which was roughly half the range for the microsections. The difference in elastic moduli observed between the micro- and cross-sections might be associated with variations in the anisotropy ratio: a more anisotropic surface appears stiffer and vice versa. This gap was not seen in adhesion forces *F_adh_*, which appeared to be similar to those in the microsections. Figure 10 shows example maps of the adhesion forces obtained for the PE7M specimen, exhibiting in detail the structural inhomogeneities introduced by precipitates on the surface of the microsection and phase boundaries between the grain aggregates on the surface of the cross-section.

## 4. Conclusions

The findings in this study led to the following observations and conclusions:

- The addition of powdered fillers in the form of montmorillonite to high-density polyethylene and aluminum hydroxide to polybutylene terephthalate resulted in a slight increase in the density of the composites obtained.
- The obtained materials were characterized by an amorphous crystalline structure. The degree of crystallinity determined by the X-ray method was about 90% for the specimens based on polyethylene and about 70% for the specimens based on polybutylene terephthalate.
- The addition of fillers to both composites resulted in a slight increase in the mean tensile strength compared to pure polymers, which at the same time led to a decrease in the strain of the specimen.
- It was found in the case of both composites that the addition of the applied fillers had little effect on the increase in the average hardness of the composites obtained.
- During the examinations conducted using atomic force microscopy, ananticorrelation was observed between the texture anisotropy and elastic modulus.

Due to the properties of the fillers, the composites obtained in the study can be used as components with increased fire resistance while not causing a large increase in the component weight and deterioration in mechanical properties compared to the pure polymer.

## Figures and Tables

**Table 1:** Phase composition of the specimens used for testing.

Specimen	Phase Composition [% by Weight]
PE	HDPE 100%
PE3M	HDPE 97% + 3% montmorillonite
PE7M	HDPE 93% + 7% montmorillonite
PBT	PBT 100%
PBT3A	PBT 97% + 3% aluminum hydroxide
PBT7A	PBT 93% + 7% aluminum hydroxide

**Table 2:** Harnesses measured by the Shore durometer and the ball indentation method.

Specimen	Mean Shore Hardness Scale D [°Sh]	Hardness, Ball Indentation HB [MPa]
PE	68 ± 4	95 ± 5
PE3M	72 ± 6	102 ± 6
PE7M	76 ± 9	105 ± 11
PBT	90 ± 5	132 ± 7
PBT3A	92 ± 9	136 ± 9
PBT7A	96 ± 12	139 ± 13

**Table 3:** Topographical and nanomechanical parameters of the surfaces of metallographic microsections: *S_q_*—RMS surface roughness, *Y_mean_*—mean Young’s pseudo-modulus, *F_adh_*—average adhesion force, *d_mean_*—mean grain size, *S_tr_*—anisotropy ratio, *D*—fractal dimension, *τ*—corner frequency.

	*S_q_* [nm]	*Y_mean_* [MPa]	*F_adh_* [nN]	*d_mean_* [nm]	*S_tr_*	*D*	*τ* [nm]
PE	2.23	582 ± 125	1.57 ± 0.14	18	0.26	2.40	59.7
PE3M	2.35	454 ± 96	2.06 ± 0.21	13	0.51	2.42	58.3
PE7M	2.82	582 ± 117	1.81 ± 0.27	15	0.25	2.39	76.1
PBT	2.77	782 ± 247	2.16 ± 0.21	20	0.31	2.46	78.7
PBT3A	6.93	1114 ± 576	3.43 ± 1.18	-	0.38	2.47	73.6
PBT7A	6.47	437 ± 141	1.68 ± 0.40	-	0.12	2.33	50.3

**Table 4:** Topographical and nanomechanical parameters of the surfaces of the fracture cross-sections: *S_q_*—RMS surface roughness, *Y_mean_*—mean Young’s pseudo-modulus, *F_adh_*—average adhesion force, *d_mean_*—mean grain size, *S_tr_*—anisotropy ratio, *D*—fractal dimension, *τ*—corner frequency.

	*S_q_* [nm]	*Y_mean_* [MPa]	*F_adh_* [nN]	*d_mean_* [nm]	*S_tr_*	*D*	*τ* [nm]
PE	6.28	171 ± 58	1.14 ± 0.30	16	0.73	2.42	69.4
PE3M	5.05	616 ± 191	2.22 ± 0.47	21	0.37	2.39	63.8
PE7M	4.53	397 ± 140	1.21 ± 0.21	17	0.52	2.41	63.3
PBT	3.08	241 ± 86	1.14 ± 0.15	16	0.46	2.44	52.0
PBT3A	8.16	292 ± 68	1.31 ± 0.19	54	0.58	2.39	51.8
PBT7A	2.57	470 ± 109	1.91 ± 0.29	18	0.56	2.37/2.59	10.7/105

## Data Availability

Data sharing is not applicable to this article.
